# Supplementary material for: Tenofovir alafenamide is superior to tenofovir disoproxil fumarate and entecavir in cost-effectiveness of treatment of chronic hepatitis B in china with new volume-based procurement policy
Source: PLoS One. 2025 Jul 11;20(7):e0327298. doi: 10.1371/journal.pone.0327298 (PMC12250602; doi:10.1371/journal.pone.0327298)
Supplement: S1 Fig — (PDF) [file pone.0327298.s001.pdf]

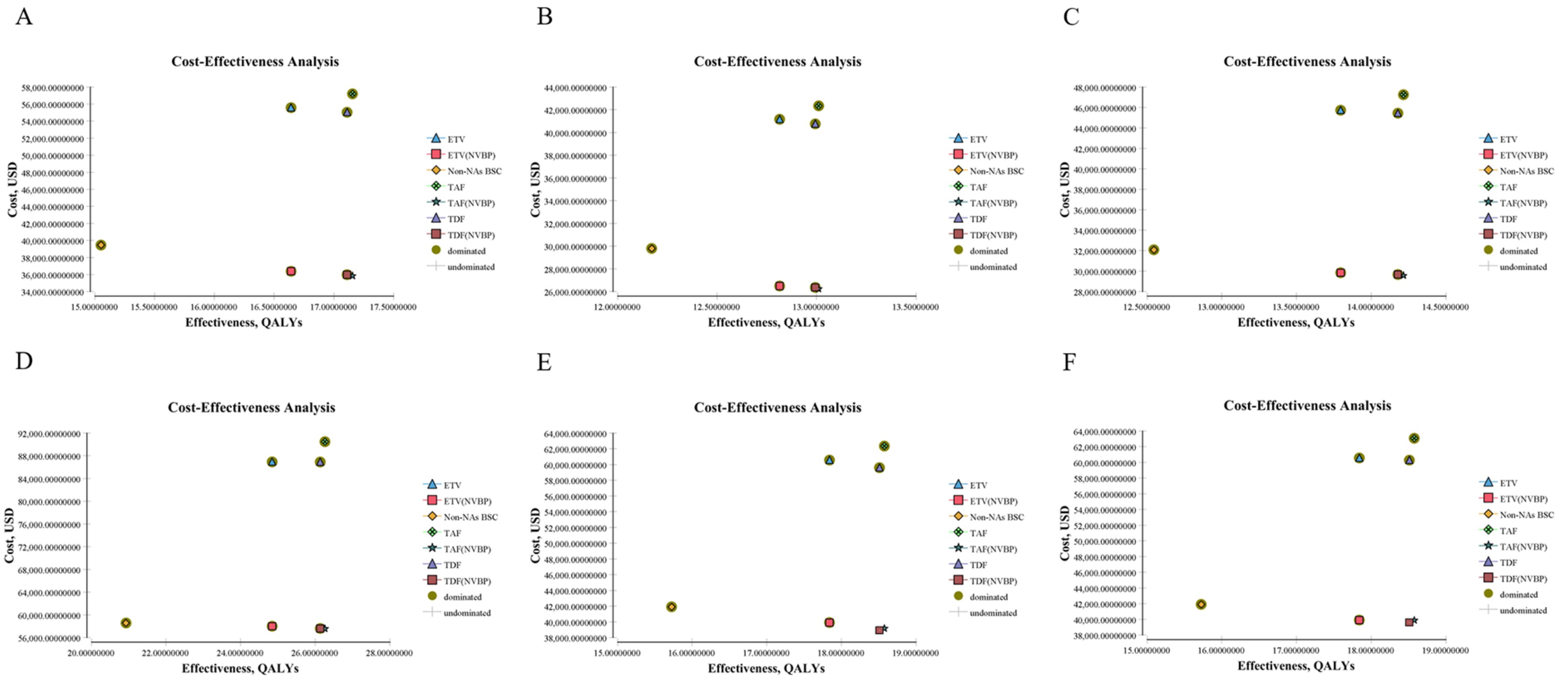

S1 Figure. Cost-effectiveness scenario analysis of various therapeutic regimens for chronic hepatitis B. A. 40-year treatment duration; B. 20-year treatment duration; C. 7% discount rate adjustment; D. 3% discount rate adjustment; E. 50% reduction in TDF/TAF monitoring costs; F. 50% increase in TDF/TAF monitoring costs.; non-NAs BSC best supportive care without nucleos(t)ide analogues, ETV entecavir, TDF tenofovir disoproxil fumarate, TAF Tenofovir alafenamide, NVBP New Volume-based purchasing.
