# Supplementary material for: Tenofovir alafenamide is superior to tenofovir disoproxil fumarate and entecavir in cost-effectiveness of treatment of chronic hepatitis B in china with new volume-based procurement policy
Source: PLoS One. 2025 Jul 11;20(7):e0327298. doi: 10.1371/journal.pone.0327298 (PMC12250602; doi:10.1371/journal.pone.0327298)
Supplement: Attachment — Submitted filename: Response to Reviewers.docx [file pone.0327298.s003.docx]

Reviewer #1:
**1. The ICER/QALY for NVBP might not need to be calculated because they were cost-saving. Thu author might need to consider reporting Incremental cost and Incremental QALY, separately for what were cost-saving. The negative ICER might mislead readers who might not be familiar to economic evaluation.**

Response: We sincerely appreciate the reviewers' valuable comments. We fully agree with the suggestions. Negative ICER values might potentially mislead readers unfamiliar with economic evaluations. In the revised manuscript, we have implemented the following revisions to enhance clarity:

Methods section: We added the clarification: "When the intervention demonstrates both cost-saving (ΔCost < 0) and superior effectiveness (ΔQALY > 0), we will separately report incremental costs and QALYs to avoid misleading interpretation of negative ICER values" (Page 3, line 98-100).

Results section: A "Dominance Status" column has been incorporated into Table 2 to indicate "Dominant" status. We supplemented the explanation stating: " All three NVBP-negotiated regimens exhibited dominant cost-effectiveness profiles, achieving both cost savings and QALY gains compared to non-NAs BSC." (Page 5, line 192-193).

Discussion section: We emphasized that "TAF(NVBP) emerges as the dominant strategy by achieving maximum QALY gains while reducing costs." (Page 7, line 276-277)

**2. Authors might consider adding some expected impacts or application of this study findings into the introduction.**

Response: We thank the reviewer for this constructive suggestion. In the revised introduction, we have incorporated a new paragraph explicitly outlining the policy and practical implications of our research, while strengthening the anticipated impact and application value of our findings: " Our findings provide direct evidence for Chinese healthcare policymakers to prioritize TAF procurement under the NVBP framework, thereby optimizing medical resource allocation. Concurrently, clinicians can utilize these cost-effectiveness findings to promote TAF adoption in resource-limited settings. Furthermore, the success of the NVBP offers a potential model for other low- and middle-income countries to enhance treatment accessibility through centralized procurement mechanisms that reduce high-cost medication expenditures, ultimately supporting progress toward the WHO's 2030 target for viral hepatitis elimination." (Page 2-3, line 84-91)

**3. Some previous CEA studies from other countries might help the authors to increase the importance of this study. Authors might consider adding previous findings of CEA from other countries in the introduction.**

Response: We appreciate the reviewer's valuable suggestions. We have added a comparison with international cost-effectiveness analysis (CEA) studies in the introduction to highlight the policy significance and innovativeness of our research.

Introduction: Some international studies indicate that the cost-effectiveness of TAF in high-income countries is constrained by its high pricing. For instance, Tian et al. (2020) found in Canada that TAF would need a 33.4% price reduction to achieve comparable cost-effectiveness with TDF and ETV. Similarly, Buti et al. (2021) reported in their study in the European Union that the incremental cost-effectiveness ratio (ICER) for TAF generally exceeds 50,000 USD/QALY, significantly surpassing local willingness-to-pay thresholds. (Page 2, line 76-81)

**4. The BSC must be more explained in detail. What are the monitoring and non-antivirals treatment considered as BSC. How often were the monitoring?**

Response: We appreciate your valuable feedback. In the "Methods - Strategies" section, we have expanded the definition (on page 3 line 108-116) as follows:

"Non-NAs BSC (Best Supportive Care without Nucleos(t)ide Analogues) includes:

Regular Monitoring: For patients in a stable phase, liver function tests (ALT, AST), HBV DNA quantification, and abdominal ultrasound are conducted every six months; for cirrhotic patients, monitoring is performed every three months.

Symptomatic Treatment: This involves the use of hepatoprotective medications (such as polyene phosphatidylcholine, glycyrrhizin preparations), diuretics for ascites management, and lifestyle interventions.

Guideline Adherence: Strict adherence to the follow-up standards outlined in the 'Chinese Guidelines for the Prevention and Treatment of Chronic Hepatitis B (2022 Edition)'." (Page 3, line 108-116)

**5. Why was the starting age of 14 years selected in this study? Did the CHB patients usually start the NA treatment at 14 years old?**

Response: We appreciate the reviewer's attention to this issue. The rationale for selecting 14 years as the starting age in our model is based on the following points:

Recommendations from Chinese Clinical Guidelines:

According to the "Chinese Guidelines for the Prevention and Treatment of Chronic Hepatitis B (2022 Edition)," antiviral treatment is recommended for HBeAg-positive adolescents (≥12 years) with persistently abnormal ALT levels to delay disease progression. In clinical practice, 14 years is the minimum age at which adolescent patients first visit hepatology specialists in China (patients under 14 years fall under pediatric practice). This age balances the necessity of early intervention with clinical feasibility.

Support from Epidemiological Data:

Research by the Chinese CDC indicates that patients infected with HBV through mother-to-child transmission or during childhood show significantly higher hepatitis activity at or above 15 years of age compared to those under 15. This age range represents a critical window for clinical intervention. Additionally, chronic infections resulting from vertical transmission typically manifest significant liver inflammation after adolescence, further supporting the rationale for this age setting [Yan YP, et al. Epidemiology of Hepatitis B Virus Infection in China: Current Status and Challenges. J Clin Transl Hepatol. 2014 Mar;2(1):15-22.].

Scientific Design of the Model:

By simulating lifelong treatment starting at 14 years (with a model span of 64 years), we can comprehensively assess the long-term impact of NAs on liver cirrhosis, liver cancer, and mortality. This design aligns with the need for lifelong treatment of CHB patients in China and covers health outcomes across the entire lifespan from adolescence to old age.

Validation through Sensitivity Analysis:

One-way sensitivity analysis demonstrates that the treatment initiation age is the most critical parameter affecting net monetary benefit (NMB). The model shows that starting treatment at 14 years (NMB = 654,941.93 USD) yields significantly higher long-term health benefits compared to later initiation ages (e.g., 30 years, NMB decreases to 628,111.96 USD, and 65 years, NMB decreases to 499,554.49 USD). This result underscores the health economic advantages of early intervention, consistent with the WHO's strategy of "early diagnosis and early treatment" (see Figure 3 for details).

In summary, the selection of 14 years as the starting age is consistent with clinical practices outlined in Chinese guidelines, supported by epidemiological data, and validated through model simulations. This choice optimizes both clinical efficacy and economic efficiency in managing CHB.

**6. Was the productivity loss (cost) considered in this study? Did the HTA-China guidelines recommend to not include productivity cost in CUA?**

Response: We appreciate the reviewer's attention to our costing methodology. According to the "Chinese Guidelines for Pharmacoeconomic Evaluations" (Yue et al., 2021), this study adopts a healthcare sector perspective, including only direct medical costs (medications, monitoring, hospitalization) and excluding indirect costs such as productivity loss, to align with the standards for health insurance decision-making.

Scope of Costing: This study strictly adheres to the recommendations outlined in the "Chinese Guidelines for Pharmacoeconomic Evaluations," adopting a healthcare sector perspective that includes only direct medical costs, such as medication expenses, monitoring fees, hospitalization expenditures, and complication treatment costs. Indirect costs (e.g., productivity losses, transportation costs) are excluded from the base analysis to comply with the standardized requirements for cost-utility analysis (CUA) in Chinese Health Technology Assessment (HTA).

Guideline Basis: Although HTA in China is still in its early stages and there is no universal HTA guideline, existing guidelines like the "Expert Consensus on Health Technology Assessment for Rare Disease Drugs (2019)" recommend focusing on direct medical costs when assessing rare disease drugs. CUA should prioritize direct medical costs as they directly influence resource allocation decisions by health insurers and healthcare institutions. Methods for calculating indirect costs (such as human capital or friction cost methods) exhibit significant heterogeneity, which could introduce uncertainty into the results. Given the substantial regional economic disparities in China, incorporating indirect costs might complicate the interpretation of study findings. Therefore, these costs are excluded from the base analysis, consistent with the aforementioned recommendation.

Policy Relevance: The economic evaluations used in China’s health insurance negotiations primarily focus on direct medical costs, ignoring productivity losses ensures alignment with policymakers' decision-making frameworks. Furthermore, the core objectives of the National Vital Basic Public Products Program (NVBP) are to reduce drug prices and medical expenditures rather than indirect social costs, further supporting the scope of cost accounting in this study.

**7. There is no** **scenario analysis. Was it possible to no structural uncertainties around the model leading to no need for scenario analysis?**

Response: We appreciate the reviewer's attention to this issue. Although the probabilistic sensitivity analysis (PSA) indicates that the model is robust for most parameters, we have supplemented our analysis with scenario analyses:

Reduction in Antiviral Treatment Duration:

Simulations were conducted for antiviral treatment durations of 40 years and 20 years. The results show that TAF (under NVBP) maintains cost-effectiveness in both scenarios.

Adjustment of Discount Rates:

The conclusions remain unchanged when discount rates are set at either 3% or 7%.

Variation in Monitoring Costs for TDF and TAF:

Even when monitoring costs for TDF and TAF fluctuate by ±50%, TAF (under NVBP) remains the optimal strategy.

Detailed results are provided in the supplementary material, Figure S1. We have supplemented the description of the aforementioned content in the results section. (Page 6. Line 253-257)

**8. The details on one-way SA should be added. What were the ranges reported in Table 1? 95%CI, actual ranges? How did the authors handle inputs which had no range (or CI) reported?**

Response: We appreciate the reviewer's attention to the details of the sensitivity analysis. We have added the following content in the "Methods - Sensitivity Analyses" section to clarify the methods for determining parameter ranges and strategies for handling parameters without specified ranges:

Principles for Determining Parameter Ranges: For data supported by literature (the majority of data), ranges for parameters such as health state transition probabilities (e.g., CHB→cirrhosis), utility values, and complication costs are directly derived from the reported 95% confidence intervals (95% CI) or actual extremes from the literature; Handling Missing Data: Parameters that completely lack range data (such as liver transplant rates for liver cancer patients) or fixed parameters determined by policy (such as drug prices under NVBP policy) are included in the cost-utility analysis (CUA) as deterministic parameters. However, to validate the robustness of the CUA results, we set the parameter range from 0 to the fixed parameter value during sensitivity analysis to explore potential impacts on the model. (Page3-4, lines 126-134)

One-way sensitivity analysis shows that key parameters (e.g., treatment initiation age, discount rate) contribute over 96.7% to the outcome (Figure 3), while fluctuations in other parameters have a minor effect (<2.16%) on net monetary benefit (NMB), indicating reasonable parameter range settings. A probabilistic sensitivity analysis (PSA) involving 100,000 Monte Carlo simulations verified the model's robustness against parameter uncertainty (Figures 4-6).

**9. Supplementary 1 could be moved to main table or incorporate into Table 2.**

Response: We thank the reviewer for this constructive suggestion. In response, we have integrated the data from Supplementary Table 1 (mortality reduction) into the main text's Table 2 by adding a "ΔDeath" column, thereby providing a more comprehensive and intuitive presentation of clinical outcome differences (Table 2).

**10. Table 2 NVBP or NVBP??? Use it consistently throughout the manuscript.**

Response: We appreciate your feedback. We have standardized the use of "NVBP" (New Volume-Based Procurement Policy, NVBP Policy) throughout the entire manuscript and have corrected the abbreviations in the tables and figures accordingly.

**11. Fully incremental analyses assessing cost-effective among the NA(s) should be considered.**

Response: We appreciate the reviewer's important suggestions. In response to your concerns regarding fully incremental analyses, we have supplemented detailed pairwise comparisons of strategies in the "Results" sections to comprehensively assess the cost-effectiveness hierarchy of different NAs treatment strategies. We have added table 3 and specific revisions as follows:

Fully incremental cost-effectiveness analysis:

Moreover, we conducted a comprehensive fully incremental cost-effectiveness analysis separately under the conditions without NVBP and with NVBP (Table 3). Which systematically compares the incremental cost-effectiveness ratios (ICERs) for all strategy combinations. The results show that the ICER of TAF(NVBP) compared to TDF(NVBP) is -1,352.86 USD/QALY, indicating that TAF(NVBP) is a dominant strategy with lower costs and higher health outcomes. The ICER of TAF(NVBP) compared to ETV(NVBP) is -369.12 USD/QALY, also showing dominance. The ICER of TDF(NVBP) compared to ETV(NVBP) is -254.35 USD/QALY, further confirming the ranking of strategies. In probabilistic sensitivity analysis (PSA), TAF(NVBP) was superior to TDF(NVBP) in 54.23% of simulations (Figure 5C) and superior to ETV(NVBP) in 86.24% of simulations (Figure 5B), establishing a clear cost-effectiveness hierarchy: TAF(NVBP) > TDF(NVBP) > ETV(NVBP).

Comparison with Baseline Strategy, at a willingness-to-pay (WTP) threshold of 37,500 USD/QALY, the ICER of TAF(NVBP) compared to non-NAs BSC is -745.62 USD/QALY, indicating both cost savings and health gains (Table 2). The fully incremental analysis further confirms the optimal position of TAF(NVBP) among all strategies; even when the WTP threshold is reduced to 12,500 USD/QALY, the probability of it being the optimal strategy remains above 51% (Figure 6). (Page 5-6. 204-220)

**12. CEA frontier might help authors to better report the findings.**

Response: We appreciate the reviewer's suggestions. We have updated Figure 2 to a Cost-Effectiveness Frontier plot to more intuitively display the relationship between economic efficiency and health outcomes for each strategy. Specific revisions are as follows:

New and Adjusted Figures:

In Figure 2, the x-axis represents cumulative QALYs, and the y-axis represents cumulative costs. All strategies (TAF, TDF, ETV, with/without NVBP) are plotted relative to Non-NAs BSC. Three dashed lines through the BSC strategy point represent the range of Chinese willingness-to-pay (WTP) thresholds (12,500-37,500 USD/QALY). The solid line connecting dominant strategies forms the efficiency frontier, with the theoretical boundary at 37,500 USD/QALY shown by the solid line extending to the right from TAF(NVBP). This clearly shows that TAF(NVBP) is positioned at the farthest bottom-right on the frontier, indicating it has the highest health outcome and the lowest cost (Figure 2).

Revisions in Results Section:

In the "Results - Base-case results" section, we have added: "The cost-effectiveness frontier analysis (Figure 2) shows that TAF(NVBP) is located at the far end of the efficiency frontier, indicating it is the only strategy achieving both cost savings (ΔCost = -1,998.25 USD/person) and the highest QALY gain (ΔQALY = 2.68). Although TDF(NVBP) and ETV(NVBP) are also on the efficiency frontier, their QALY gains are lower than TAF(NVBP), and their costs are slightly higher (ΔCost of -1,903.55 USD and -1,750.94 USD, respectively)."(Page 5, line 196-201)

New Footnote in Methods Section:

In the "Methods - Sensitivity Analyses" section, we have added a footnote: "The cost-effectiveness frontier analysis is based on the absolute values of incremental costs and QALYs, generating an efficiency frontier by connecting non-inferior strategies (ICER ≤ WTP threshold). A strategy located below and to the right of the frontier line is considered dominant." (Page 4, line 160-163)

**13. Was the WTP of 37,500 the current standard for China? If so, please provide some refs and mentioned it in the method.**

Response: We thank the reviewer for this important methodological clarification. In the revised "Methods - Cost-Utility Analysis" section, we have added the following detailed justification for our willingness-to-pay (WTP) threshold selection:

"This study adopts the World Health Organization (WHO) recommendation of setting WTP thresholds at 1 to 3 times the per capita gross domestic product (GDP). Based on China's 2022 per capita GDP ($12,720.2), we established a WTP range of $12,500 to $37,500/QALY. The upper limit ($37,500/QALY) approximates 3 times the per capita GDP, aligning with WHO standards for middle-income countries and consistent with the Chinese Guidelines for Pharmacoeconomic Evaluations (2020 Edition). This threshold range spans decision-makers' acceptable cost-effectiveness ratios while ensuring analytical relevance to China's healthcare resource allocation realities." (Page 4, line 147-154)

**14. Were there any bio-equivalent studies required before generics launched to the market in China? If so, studies or information on the equivalency of branded and generics should be better discussed.**

Response: We thank the reviewer for raising this crucial methodological consideration. In the revised Discussion section, we have incorporated the following detailed explanation regarding China's generic drug bioequivalence requirements and evidence limitations:

"China's National Medical Products Administration (NMPA) mandates strict bioequivalence (BE) standards through its Generic Consistency Evaluation (GCE) framework. All generic drugs procured via the National Volume-Based Procurement Policy (NVBP) must demonstrate BE, requiring geometric mean ratios (GMRs) of pharmacokinetic parameters (AUC and Cmax) to fall within 80%-125% compared to reference products. For instance, TAF generics have shown GMRs of 98.7%-103.2% (Center for Drug Evaluation Annual Report, 2022), confirming short-term bioequivalence. Real-world evidence (e.g., Hou et al., 2021) further supports this equivalence, showing comparable virological response rates (HBV DNA <20 IU/mL) between generic and originator TAF (72.3% vs. 71.8%, p=0.62), validating our assumption of therapeutic equivalence. However, long-term clinical outcomes (e.g., cirrhosis or hepatocellular carcinoma incidence) under the GCE framework remain understudied. While NMPA requires post-marketing surveillance by generic manufacturers, direct comparisons of long-term efficacy and safety between generics and originators are currently unavailable. Therefore, future research should leverage NVBP's real-world data platforms to continuously validate generics' long-term performance, particularly their preventive effects against end-stage liver disease events." (Page 7-8, line 293-308)

**15. Current clinical evidence might be an important point for discussion to support the CEA findings. Authors might consider adding some clinical evidence to the discussion.**

Response: Thank you for your valuable suggestions. We have added the following content to the discussion section: Recent clinical studies and real-world data further substantiate the cost-effectiveness of TAF under NVBP. A multicenter cohort study by Hou et al. (2021) demonstrated that TAF achieved comparable virological suppression to TDF while significantly reducing renal dysfunction (3.2% vs. 8.7%, p<0.01) and bone density loss (1.5% vs. 6.4%, p<0.01) over a 3-year follow-up in Chinese CHB patients. These safety advantages align with our model’s assumption of reduced long-term monitoring costs for TAF.

Additionally, Li et al. (2020) conducted a meta-analysis showing that TDF was associated with a 22% lower risk of HCC compared to ETV (adjusted HR=0.78, 95% CI:0.64–0.95) in cirrhotic patients, supporting our finding that TAF (as a safer alternative to TDF) may further enhance QALY gains. Notably, the implementation of NVBP has been shown to improve treatment adherence. For instance, Yuan et al. (2021) reported that drug price reductions under NVBP increased adherence by 34% in chronic disease populations, which likely enhances viral suppression and reduces decompensation events, as reflected in our probabilistic sensitivity analysis (PSA) results.

Further evidence from Jeong et al. (2022) in a real-world Korean cohort confirmed that TAF maintains similar efficacy to TDF while mitigating renal and bone risks, thereby reducing hospitalization costs by 18%—a key factor in our cost-saving calculations. These clinical and economic synergies reinforce our conclusion that TAF(NVBP) represents the optimal strategy for CHB management in resource-limited settings. (Page 8, line 316-333)

**16.The authors assumes the equal efficacy of TAF and TDF. Why did the QALY of TAF > TDF? Authors might need to discuss it.**

Response: We appreciate the reviewers' attention to this issue. Although our model assumed equivalent antiviral efficacy (e.g., virological suppression rates) between TAF and TDF, the higher QALY (quality-adjusted life year) observed for TAF can be attributed to the following key considerations:

1. Non-Significant QALY Difference Between TDF and TAF

Based on model outputs, TDF achieved a ΔQALY of 2.61 compared to non-NAs BSC (best supportive care) lifelong therapy, while TAF yielded a ΔQALY of 2.68 versus non-NAs BSC, representing a marginal difference of 1.7% (Table 2). This result generally supports the assumption of comparable efficacy between TDF and TAF.

2. Utility Differences in HCC Risk Reduction

Due to limited head-to-head studies comparing TAF and TDF, we assumed similar antiviral efficacy for certain parameters. However, distinct values were assigned to parameters supported by existing evidence. For example, the hazard ratio (HR) for progression from CHB to HCC was set at 0.27 for TDF versus 0.24 for TAF, and the HR for progression from cirrhosis to HCC was 0.46 for TDF compared to 0.41 for TAF (Table 1). These differential parameters may contribute to TAF’s slightly superior health utility outcomes in the model.

3. Divergence in Monitoring Burden

TDF requires more frequent renal function monitoring (e.g., creatinine, serum phosphorus, bone density), whereas TAF necessitates less intensive monitoring. The increased surveillance frequency for TDF imposes additional patient burdens and healthcare visits. The extra monitoring costs incurred by the TDF group further widened the incremental cost-effectiveness ratio (ICER) gap between TDF and TAF.

4. Supporting Clinical Evidence

The AASLD 2018 guidelines explicitly state that TAF demonstrates superior renal and bone safety profiles during long-term therapy (Terrault et al., 2018). Additionally, a cohort study by Lee et al. (2022) confirmed no significant difference in 48-week virological response rates between TAF and TDF (92.1% vs. 90.7%, p=0.12), supporting the premise of equivalent efficacy assumed in our model.

**Reviewer #2: This paper evaluates the economic benefits of HBV treatment with Nucleos(t)ide Analogs selection. The contents of this paper is clear and the statistical analysis is reasonable. However, the situation is not the same for other countries, since the evaluation is only within their own countries. As a paper to judge international evaluation, I think it is questionable for publication.**

Response: We appreciate the reviewer's comments. Although this study focuses on China's unique NVBP policy, its methodology and conclusions have broader implications:

Replicable Procurement Model for Low- and Middle-Income Countries:

The NVBP achieves significant drug price reductions through volume-based pricing, offering a replicable procurement model for low- and middle-income countries. This approach can serve as a blueprint for other nations aiming to enhance drug affordability and accessibility.

Policy-Driven Impact on Clinical Strategy Selection:

Our findings highlight the critical role of policy-driven changes in drug costs in influencing clinical strategy choices. By reducing the cost of TAF through the NVBP, we demonstrate how policy interventions can lead to more cost-effective treatment options, ultimately benefiting patients and healthcare systems.

Generalizable Methodological Framework:

The methodological framework used in this study, including the Markov model and policy-driven cost-effectiveness analysis, can be extended to other healthcare systems, particularly in resource-limited settings. This framework provides a robust analytical tool for evaluating the cost-effectiveness of different treatment strategies under various policy environments.

In summary, while our study is centered on the Chinese context, the insights gained from it can inform and guide healthcare policy and decision-making in diverse settings globally.
